# Extra-Uterine Pregnancy of Four Years’ Standing, the Patient in the Interim Being Twice Delivered of a Healthy Living Child

**Published:** 1856-08

**Authors:** A. W. Heise

**Affiliations:** Addison, Ill.


*Extra-Uterine Pregnancy of Four Years' Standing, the patient in the interim being twice delivered of a healthy living child.* By A. W. Heise, M.D., of Addison, Ill.

In November, 1855, I was called to visit Mrs. Yungels, residing one mile east of Aurora, and thirty miles west of this place. Upon my arrival I found the patient, a woman of robust constitution, 36 years of age, to have been 10 days since delivered of a healthy male child. The three or four days following delivery she was quite well, since which time she has had a chill every day, followed by fever and profuse perspiration. Has had the usual lochial discharge, but no secretion of milk; pulse 100 to 110; tongue red, dry, and hard; no appetite. On applying my hand to the abdomen, which is painful and irritable, I find an enlargement resembling a hard tumor, which is moveable and not connected with the integuments, commencing in the umbilical region about three inches above the umbilicus, extending downward parallel with the linea alba to the os pubes, filling ¾ of the lumbar and iliac region of the right side. Left of the linea alba it seems to be perfectly free from any morbid growth. Examination per vag. shows the uterus in situ, contracted; the os uteri somewhat enlarged, hot, dry, and painful; vagina natural. By moving the uterus and placing the other hand over the tumor, the motion of uterus affects the tumor, and *vice versa.*

Upon inquiry I was informed, that four years since, the patient supposed herself pregnant, experiencing the usual symptoms attendant upon gestation for a period of 10 months, during which time the abdomen constantly enlarged, particularly the right side, which was hard and painful, rendering her unable to lie upon it after the third month. At the end of 10 months labor commenced, and a midwife was summoned, who after expressing her fears that the child did not "lay right,"

declared it was yet out of her reach, the os uteri not at all dilated, would not be delivered yet, &c.

Bearing-down pains however increased, and were finally relieved by a discharge of a large quantity of watery matter, having the appearance of beef brine, followed by coagulated blood. This not only very much relieved the patient, but diminished the size of the abdomen, and the midwife assured her she had suffered only from obstructed menstruation, and would soon recover. The abdomen gradually decreased in size for the space of three weeks, when the lochial discharge ceased, and with it the diminishing of the bowels, leaving still an enlargement of the right side which she was yet unable to lie upon.

Finding no alteration in the tumor from that time, four months afterwards she consulted a physician, who told her he could not ascertain the nature of the tumor; could do nothing for her without an operation, which, so long as she suffered so little pain, and it did not enlarge, he would not advise.

She soon became *enciente*, and in time was delivered of a large healthy child. Parturition was easy, and she soon regained her usual health, having experienced no unusual symptoms, with the exception of the total absence of any secretion of milk. The tumor had been painful during confinement, but otherwise retained its former appearance.

Eighteen months afterwards, she again became pregnant, and in November, 1855, was once more delivered of a healthy male child—both are now living.

Dr. Young, of Aurora, who attended her says, he observed nothing unusual in her case, did not notice any enlargement of the abdomen: called again, and discharged her as doing well.

On the 10th day after confinement I first saw the patient, and found her as above stated.

I prescribed those medicines which were indicated, directed emolient poultice to the abdomen, and left, with directions that if the patient did not improve, or there was any alteration in the tumor, to inform me. Thought the tumor might be an enlargement of the right ovarium, and that when the irritation of the uterus subsided and the fever abated it would cease to be painful, and she might again enjoy her usual health.

Heard no more from her until May 5, 1856. I was summoned to see her again. I found her much emaciated, exhibiting a great degree of nervous excitability, pulse 90, small and irritable—has suffered much from pain since I saw her, having been constantly confined to her bed. The tumor at the umbilicus has ulcerated, and discharges a very offensive fluid. Through the orifice, which is nearly the size of a half dime, a bone has protruded, another now closes the opening.

The case was now plain, and I advised an immediate operation. Dr. Young, of Aurora was called to assist, and upon his arrival, requested to have his friend, Dr. Allaire, of Aurora, present.

Adhesion of the peritoneum had taken place around the orifice to the extent of from three to three and a half inches. I made an incision of about three inches towards the right lumbar region, and commenced at once to extract the bones. The flesh was decomposed, but the skeleton was perfect and was of the size of a fœtus at the seventh month, though the bones appeared to have the firmness of two years' growth. Its extraction through the rather small orifice was tedious, in which Dr. Allaire very kindly and effectually assisted, Dr. Young, meantime, quieting the patient by administering chloroform. The bones of the head and pelvis were too large to pass through the incision, until they were severed by a strong pair of scissors. We succeeded in removing all the bones, together with a mass of semi-decomposed matter from the sac. The sac, which was formed of a grisly substance, and so hard as to almost resist the passing of the bistoury, seemed to have contracted close around the fœtus, and evidently had performed the office of placenta and uterus, it being connected with the latter.

The wound was simply dressed with lint, a bandage applied, and the patient directed to lie in a position to facilitate the discharges. She was left under the care of Dr. Young, and up to May the 10th, was doing well.

